# Zebrafish knock-ins swim into the mainstream

**DOI:** 10.1242/dmm.037515

**Published:** 2018-10-24

**Authors:** Sergey V. Prykhozhij, Jason N. Berman

**Affiliations:** 1 Department of Pediatrics, Dalhousie University, IWK Health Centre, Halifax, NS B3K 6R8, Canada; 2 Department of Microbiology and Immunology, Dalhousie University, Halifax, NS B3H 4R2, Canada; 3 Department of Pathology, Dalhousie University, Halifax, NS B3H 4R2, Canada

**Keywords:** CRISPR/Cas9, Genome editing, Point mutations, Zebrafish

## Abstract

The zebrafish is an increasingly popular model organism for human genetic disease research. CRISPR/Cas9-based approaches are currently used for multiple gene-editing purposes in zebrafish, but few studies have developed reliable ways to introduce precise mutations. Point mutation knock-in using CRISPR/Cas9 and single-stranded oligodeoxynucleotides (ssODNs) is currently the most promising technology for this purpose. Despite some progress in applying this technique to zebrafish, there is still a great need for improvements in terms of its efficiency, optimal design of sgRNA and ssODNs and broader applicability. The papers discussed in this Editorial provide excellent case studies on identifying problems inherent in the mutation knock-in technique, quantifying these issues and proposing strategies to overcome them. These reports also illustrate how the procedures for introducing specific mutations can be straightforward, such that ssODNs with only the target mutation are sufficient for generating the intended knock-in animals. Two of the studies also develop interesting point mutant knock-in models for cardiac diseases, validating the translational relevance of generating knock-in mutations and opening the door to many possibilities for their further study.

## Abstract (precis)

**Summary:** With the co-publication of three research articles describing CRISPR/Cas9-based approaches for knocking in mutations in zebrafish (dmm035352, dmm035469 and dmm035972), this Editorial reflects on the current state of genome editing for disease modeling in zebrafish.

## Introduction

One of the great potentials of zebrafish (*Danio rerio*) is to generate accurate models of human genetic diseases to recapitulate their clinical features, to understand the molecular mechanisms that underpin them, and to model treatments and disease management approaches. Before the advent of precise genome editing, understanding a missense mutation within a protein-coding human gene in zebrafish necessitated designing an mRNA expression vector or a transgenic construct. These approaches did not actually replace the normal (wild-type) zebrafish gene with an altered human one. Additionally, expression of a human (trans)gene raises the question whether that gene is, in fact, functional in fish. Does the mutant gene function in fish? Are expression levels sufficient? Is expression in the right place and at the right time? These answers may be elusive.

Imagine instead the ability to map an amino acid in a protein from another species to a specific zebrafish protein residue and then mutate it. This is the essence of point mutation knock-in; namely, the replacement of wild-type nucleotides with mutant ones by inducing endogenous recombination with genome-editing reagents. An investigator can then confidently know that his or her gene of interest is modified precisely and expressed in the biologically relevant way – under the control of the endogenous promoter. With this refined approach, the mutation is the only variable under study. One potential disadvantage of current knock-in methodologies is that they cannot be used directly for introducing dominant and lethal mutations, which might be better studied using transgenic approaches that afford inducibility.

**Fig. 1.**
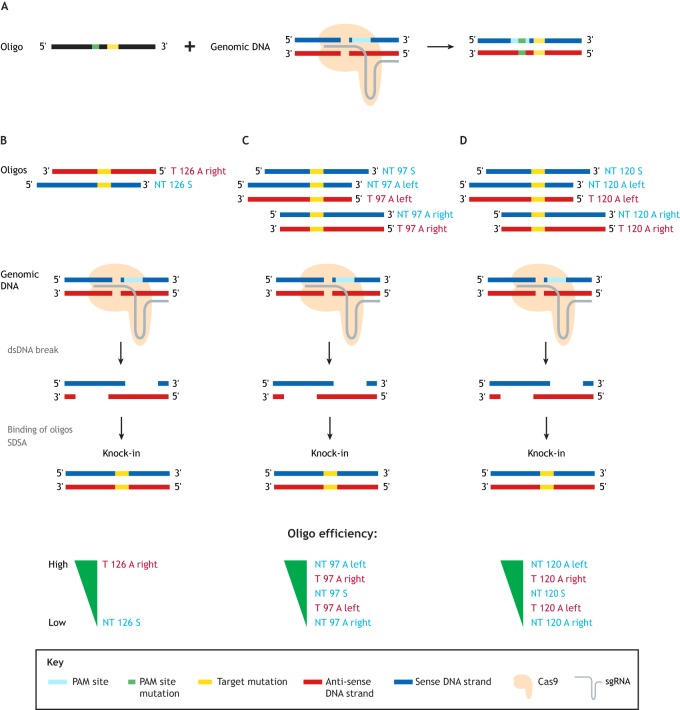
**Comparison of different study results using oligos for point mutation knock-ins.** The figure panels show the Cas9-sgRNA CRISPR complex cutting genomic DNA and subsequent homology-directed repair by resection and knock-in mutation insertion. Synthesis-dependent strand-annealing (SDSA) is the DNA repair process involved in generating knock-ins when an ssODN (oligo) is present. (A) The basic strategy of point mutation knock-ins. The first step includes the identification of a functional sgRNA to couple with the Cas9 nuclease and direct it to the genomic site of choice. Second, the donor oligo with the mutation of interest and mutation(s) in sgRNA site or PAM is designed. Mutating the sgRNA homology site and/or the PAM site prevents subsequent rounds of Cas9-induced cuts of the edited genomic site. Third, upon the Cas9-induced break in genomic DNA, homology-dependent repair using the provided oligo can occur and the mutation is inserted into the genome. (B) The results of studies employing a comparison of ‘NT 126 S’ (sense symmetric) and ‘T 126 A right’ (anti-sense asymmetric) oligo knock-in efficiencies in zebrafish and *in vitro* (Prykhozhij et al., 2018b; Richardson et al., 2016). (C) The results of a cell culture study demonstrating which types of asymmetric oligos are more efficient (Liang et al., 2016). (D) The results of the study in this issue (Boel et al., 2018) that shows that symmetric oligos such as ‘NT 120 S’ perform nearly as well as two of the asymmetric oligos (‘NT 120 A left’ and ‘T 120 A right’), which, in turn, perform much better than their counterparts ‘NT 120 A right’ and ‘T 120 A left’.

Generation of precise point mutations in zebrafish for basic research and disease modeling studies is an important contribution of the burgeoning CRISPR/Cas9 technology, which uses a single-guide RNA (sgRNA) molecule to guide the Cas9 endonuclease to the genomic site of choice. Single-stranded oligodeoxynucleotides (ssODNs), or oligos for short (as referred to herein), are currently the donor templates of choice for mutation knock-ins (Fig. 1A). These oligos can vary in length and be either ‘target’ (T) or ‘non-target’ (NT) in orientation, and have either symmetric or asymmetric homology arms. NT oligos correspond to the strand not bound by the sgRNA, which contains the protospacer-adjacent motif (PAM) sequence; hence, the oligos are sometimes referred to as ‘sense’. Conversely, T oligos are derived from the strand bound by the sgRNAs and are correspondingly often referred to as ‘anti-sense’.

Our own group previously reviewed zebrafish studies aimed at developing precise point mutation techniques using CRISPR/Cas9 (Prykhozhij et al., 2018a), including the first successful zebrafish study on germline transmission of a point mutation knock-in (Armstrong et al., 2016). In this Editorial, we will explore the implications of three papers published in this issue of Disease Models & Mechanisms that demonstrate point mutation knock-ins in zebrafish, and the advantages and challenges of working with such precise mutants (Boel et al., 2018; Tessadori et al., 2018; Farr et al., 2018). These papers pave the way toward CRISPR-based oligo knock-ins becoming widely acceptable as the preferred approach to generate point mutations in zebrafish.

## The focus on technology

The paper by Boel et al. focuses on the technical aspects of knock-in optimization (Boel et al., 2018). The first key observation in the Boel paper is that using oligos to guide homology-directed repair (HDR) is quite error prone. Although this has been previously shown in zebrafish (Burg et al., 2016; Hruscha et al., 2013; Hwang et al., 2013; Prykhozhij et al., 2018b), this report does so much more systematically. The authors identified typical ‘knock-in with indel’ events, insertions of partial and multiple oligos, inverse oligo insertions and other abnormal events. The prevalence of these abnormal events ranged from a few percent to >50% of the total knock-in events, thereby reducing the correct knock-in rates from 4-8% (total knock-ins) to 1-4% (correct). This result suggests that caution and routine use of next-generation sequencing (NGS) approaches are needed to assess the error rate of any new knock-in strategy. Given that each oligo in this study contained six nucleotide changes, the authors examined how much the distance between the Cas9-induced cut site and mutation influences the knock-in efficiency. Predictably, increasing this distance resulted in decreased knock-in efficiency, which was more pronounced for oligos with shorter homology arms, such as 60 nucleotides (nt), and for asymmetric (A) oligos, in which, for example, the left homology arm is 30 nt and the right is 90 nt long. This is consistent with previous studies (Paquet et al., 2016), and suggests that researchers use longer or symmetric (S) homology arms for engineering knock-ins in which the mutation site is at large distances from the Cas9-induced cut sites.

Boel and colleagues also investigated whether asymmetric homology arm design for 120 nt oligos can improve knock-in efficiency (Boel et al., 2018). No single type of oligo design performed best in their system, so they concluded that homology arm asymmetry is likely not a general strategy for improving knock-in rates. This somewhat contradicts previous studies in cell culture systems that found target asymmetric ‘T 126 A right’ oligos superior to non-target symmetric ‘NT 126 S’ oligos (Fig. 1B) (Richardson et al., 2016), and that ‘NT 97 A left’ or ‘T 97 A right’ were both superior to the symmetric ones (Fig. 1C) (Liang et al., 2017).

In zebrafish, our group recently confirmed the *in vitro* findings of Richardson et al. (2016) that target asymmetric oligos perform significantly better than the non-target symmetric oligos for two different knock-ins into the *tp53* gene (Fig. 1B) (Prykhozhij et al., 2018b). Boel and colleagues also note, however, that for three out of the four sites in the zebrafish genome they targeted, the combined knock-in rates for 120 nt asymmetric (‘NT 120 A left’ and ‘T 120 A right’) oligos were higher than those of symmetric ones. These 120 nt asymmetric oligos also performed consistently better than the ones with opposite asymmetries (‘NT 120 A right’ and ‘T 120 A left’) (Fig. 1D), which is consistent with the known properties of homologous recombination-based DNA repair by resection and synthesis-dependent strand annealing (Paix et al., 2017). The authors make a reasonable recommendation to test these oligos in parallel with symmetric ones. Boel et al.’s main aim was technical knock-in optimization. The oligos they tested contained six nucleotide mismatches and were not intended to change the amino acid sequence of the protein product (Boel et al., 2018). The oligos used in our study contained non-silent disease-relevant mutations, which is likely a more common scenario for mutation knock-in studies. Targeted cells might have different tolerance for silent and non-silent mutations, which can result in different frequencies of successfully targeted clones. Future efforts will either resolve these efficiency discrepancies or perhaps render them irrelevant due to further improvement of genome-editing technologies.

## Modeling diseases via knock-ins with short oligos

Large-scale sequencing technology has greatly facilitated the identification of novel potentially disease-causing genomic variants. We have yet to understand the functional implications of these variants, but zebrafish can assist us in clarifying the complex genetic and molecular underpinning of these variants. The zebrafish has become one of the most sought after organisms in which to generate mutants that can serve as models of diseases caused by specific genomic variants. Most of these variants cannot be accurately modeled by complete gene inactivation or knockout and require knock-in approaches to introduce a specific mutation. Despite recent technological advances, few disease-associated zebrafish point mutants have been generated to date. The papers by Farr et al. and Tessadori et al. in this issue of Disease Models & Mechanisms describe point mutation knock-ins of several genetic variants implicated in inherited cardiac diseases (Farr et al., 2018; Tessadori et al., 2018). Tessadori et al. focus on mutations found in Cantú syndrome, a rare disease characterized by multiple cardiac abnormalities, bony changes and hair thickening. Farr et al. describe a zebrafish model of a *PBX3* A136V mutation, which is enriched in a subset of congenital heart defects.

Both studies employed *in vitro*-transcribed sgRNA and either nCas9n mRNA (Jao et al., 2013) or Cas9 protein (Tessadori et al., 2018), together with oligos encoding the modifications. The distances between the Cas9 cut site and the nucleotide(s) to be mutated were 0-5 nt in the paper by Tessadori et al. and 10 nt in the paper by Farr et al. Both distances were in the previously reported optimal range (Paquet et al., 2016). Importantly, the mutations overlapped the sgRNA binding sites. Tessadori et al. used non-target strand mutant oligos with 25 nt homology arms and Farr et al. tested both non-target and target oligos, which were asymmetric (35 nt and 15 nt homology arms) if counted from the cut site. Interestingly, Farr and colleagues found that oligos with 25 nt homology arms worked well, whereas oligos with 20 nt and 40 nt homology arms failed. It is not clear whether homology arm asymmetry accounts for the performance differences, but it is likely that the shorter 10 nt homology arms are generally too short and 50 nt homology arms oligos might be too long for HDR. Once knock-in reagents were injected and the fish were subsequently bred, the resulting knock-in alleles needed to be detected and verified. Both papers employed Sanger sequencing and restriction enzyme digestion of the targeted site to evaluate knock-ins. Alternative approaches, such as allele-specific PCR, could be a more broadly applicable option (Prykhozhij et al., 2018b).

Both groups leveraged cut site proximity to the mutation sites and undertook some oligonucleotide size optimization to achieve efficient knock-in generation and germline transmission. The approaches described in these papers might prove effective for other mutation knock-ins in which the desired change is proximal to the Cas9 cut site. However, longer homologies and asymmetric homology arm designs might be needed for targeting mutation sites that are more distant from the predicted Cas9 cut site. Boel et al. (2018) suggest that much of the increase in efficiency observed due to lengthening oligos from 60 nt to 120 nt comes from aberrant repair events. It is therefore possible that 50-60 nt is a ‘sweet spot’ oligo length for cut-site proximal target mutation. The Farr and Tessadori groups should be lauded for identifying the minimal effective oligo size and simple knock-in procedures.

The main aim of these two studies was to model heart disease in zebrafish. Therefore, the mutations the authors introduced needed to produce a tractable, disease-relevant phenotype. Farr et al. aimed to test whether the zebrafish *pbx4* A131V variant (which is homologous to the human *PBX3* A136V mutation) could function as a modifier allele, resulting in a congenital heart defect. The *pbx4* A131V allele did not produce a clearly discernable phenotype, but it was also not completely functional because its presence increased the severity of heart defects when combined with a null *pbx4* allele (Farr et al., 2018). Tessadori and colleagues identified a convincing phenotype for their heterozygous and homozygous *kcnj8* V65M Cantú syndrome mutants. Their *abcc9* G989E knock-in had a phenotype similar to that of the *kcnj8* V65M knock-in mutants, confirming a more generalized genotype-phenotype correlation (Tessadori et al., 2018). These newly developed zebrafish models could be used to improve our understanding of genetic heart disease and to test therapeutic approaches.

## Conclusions

In sum, these three papers highlight various technical optimizations that can achieve robust and reproducible knock-in mutations, leading to zebrafish models with greater fidelity to the human diseases they are modeling. Lessons from these papers will be instructive to other investigators by providing important factors to consider when designing CRISPR/Cas9-based knock-ins in zebrafish. Although the generation of knock-in mutations continues to pose challenges, its successful implementation promises to be of tremendous value to the broader model organism community to study complex genetics that contribute to disease, in genes and in non-coding regions of the genome. By incorporating these mutants into high-throughput drug screening pipelines, the zebrafish holds great potential to provide rapid, cost-effective preclinical therapeutic data in a uniquely whole-organism vertebrate context that can streamline confirmatory murine studies and ultimately inform future clinical trials for patients with genetic disorders.
